# Supplementary material for: From sequence to enzyme mechanism using multi-label machine learning
Source: BMC Bioinformatics. 2014 May 19;15:150. doi: 10.1186/1471-2105-15-150 (PMC4229970; doi:10.1186/1471-2105-15-150)
Supplement: Additional file 2 — Java code of ml2db. Additional file ml2db_code.tar.gz contains the Java source code to run the multi-label machine learning experiments and save the results to database. The code’s Javadoc is included. [file 1471-2105-15-150-S2.zip › additional file 2/ml2db/ml2db/doc/mulan/data/LabelPairsDependenceIdentifier.html]

LabelPairsDependenceIdentifier


JavaScript is disabled on your browser.


- Overview
- Package
- Class
- Use
- Tree
- Deprecated
- Index
- Help

- Prev Class
- Next Class

- Frames
- No Frames

- All Classes

- Summary:
- Nested |
- Field |
- Constr |
- Method

- Detail:
- Field |
- Constr |
- Method


mulan.data

## Interface LabelPairsDependenceIdentifier

- All Known Implementing Classes:
  :   ConditionalDependenceIdentifier, UnconditionalChiSquareIdentifier

  ---

    

  ```
  public interface LabelPairsDependenceIdentifier
  ```

  An interface for various types of dependency identification between pairs of labels.
  .

  Version:
  :   30.11.2010

  Author:
  :   Lena Chekina (lenat@bgu.ac.il)

- - ### Method Summary

    Methods

    | Modifier and Type | Method and Description |
    | --- | --- |
    | `LabelsPair[]` | `calculateDependence(MultiLabelInstances mlInstances)` Calculates dependence level between each pair of labels in the given multilabel data set |
    | `double` | `getCriticalValue()` Returns a critical value |

- - ### Method Detail


    - #### calculateDependence

      ```
      LabelsPair[] calculateDependence(MultiLabelInstances mlInstances)
      ```

      Calculates dependence level between each pair of labels in the given multilabel data set

      Parameters:
      :   `mlInstances` - multilabel data set

      Returns:
      :   an array of label pairs sorted in descending order of pairs' dependence score


    - #### getCriticalValue

      ```
      double getCriticalValue()
      ```

      Returns a critical value

      Returns:
      :   critical value


- Overview
- Package
- Class
- Use
- Tree
- Deprecated
- Index
- Help

- Prev Class
- Next Class

- Frames
- No Frames

- All Classes

- Summary:
- Nested |
- Field |
- Constr |
- Method

- Detail:
- Field |
- Constr |
- Method
